# GLS and GLS2 Glutaminase Isoenzymes in the Antioxidant System of Cancer Cells

**DOI:** 10.3390/antiox13060745

**Published:** 2024-06-20

**Authors:** Juan De los Santos-Jiménez, José A. Campos-Sandoval, Francisco J. Alonso, Javier Márquez, José M. Matés

**Affiliations:** 1 Canceromics Lab, Departamento de Biología Molecular y Bioquímica, Universidad de Málaga, 29071 Málaga, Spain; jsj@uma.es (J.D.l.S.-J.); jacs@uma.es (J.A.C.-S.); fcarrion@uma.es (F.J.A.); marquez@uma.es (J.M.); 2 Instituto de Investigación Biomédica de Málaga (IBIMA-Plataforma BIONAND), Universidad de Málaga, 29590 Málaga, Spain

**Keywords:** cancer, ferroptosis, glutaminase, glutaminolysis, glutathione, metabolic reprogramming, oxidative stress, ROS

## Abstract

A pathway frequently altered in cancer is glutaminolysis, whereby glutaminase (GA) catalyzes the main step as follows: the deamidation of glutamine to form glutamate and ammonium. There are two types of GA isozymes, named GLS and GLS2, which differ considerably in their expression patterns and can even perform opposing roles in cancer. GLS correlates with tumor growth and proliferation, while GLS2 can function as a context-dependent tumor suppressor. However, both isoenzymes have been described as essential molecules handling oxidant stress because of their involvement in glutathione production. We reviewed the literature to highlight the critical roles of GLS and GLS2 in restraining ROS and regulating both cellular signaling and metabolic stress due to their function as indirect antioxidant enzymes, as well as by modulating both reductive carboxylation and ferroptosis. Blocking GA activity appears to be a potential strategy in the dual activation of ferroptosis and inhibition of cancer cell growth in a ROS-mediated mechanism.

## 1. Introduction

Glutaminase (GA, EC 3.5.1.2) is the enzyme responsible for catalyzing the conversion of glutamine (Gln) to glutamate (Glu) and represents the first step in glutaminolysis [1]. Gln has essential functions in cancer, providing amino acids, lipids, nucleotides, hexosamines, and polyamines but also rendering metabolic energy in the form of adenosine triphosphate (ATP) or being used as a pleiotropic cell signaling molecule [2]. In addition, Gln is indispensable for synthesizing reduced glutathione (GSH), the most abundant intracellular antioxidant molecule and the main player in the GSH–antioxidant system, formed by GSH, oxidized glutathione (GSSG), glutathione reductase (GR), and glutathione peroxidases (GPXs) [3]. While Glu affords resources for GSH synthesis, the limited oxidation of Gln through glutaminolysis restricts oxidative phosphorylation (OXPHOS) and mitigates reactive oxygen species (ROS) levels that are otherwise harmful to cells [2]. Nonetheless, ROS can cause damage to cancer cells when levels are excessive, but, indeed, they are a needed signaling mechanism for tumor homeostasis [4]; hence, ROS levels are precisely regulated by a set of antioxidant enzymes, including, indirectly, the function of GA isoenzymes [1]. The key role of antioxidant systems and the balance of ROS can be exploited for the design of novel strategies to target cancer [2,3], which is discussed in this review.

## 2. Glutaminase Isoenzymes in the Control of Cancer Redox Homeostasis

The metabolic alterations observed in cancer are a consequence of the dysregulation of the expression of metabolic enzymes such as GA isoenzymes [5]. In mammals, including humans, the *GLS* gene encodes two isoforms, the largest kidney-type glutaminase (KGA) and glutaminase C (GAC), the latter usually being the variant most frequently overexpressed in cancers. Both isoforms can be collectively called GLS isoenzymes [6]. On the other hand, the *GLS2* gene encodes two more isoforms, glutaminase B (GAB) and the shorter liver-type glutaminase (LGA), and both can be referred to as GLS2 isoenzymes [7]. The secondary functions of these metabolic enzymes are linked with several molecular mechanisms affecting cancer, being sharply different for GLS and GLS2. Therefore, GAs are related to both cancer progression or antitumoral effects by altering the signaling, regulation, and oxidative status of cancer cells (Table 1 and Table 2). 

GLS2 has been related to both tumor suppression properties and prooncogenic effects. Thus, its role in cancer is considered to be context-dependent and has been thoroughly reviewed recently [42]. However, its function as an oncogenic factor or a tumor suppressor has been related, at least partially, to its ability to modulate antioxidant capacity [1]. In the nucleus, GLS2 also participates in redox regulation and the growth-arrest program, interacting with key factors [5,7]. Although human GLS2 has structural determinants for mitochondrial targeting, and it does not possess a typified nuclear localization signal, its nuclear translocation has been reported and it can work as an indirect transcription factor regulating gene expression and modulating other nuclear proteins [43]. In fact, a mechanism implying its tumor suppressor ability is related to p53 and was first characterized in 2010 [44,45]. GLS2 expression was found to be induced by p53 and evoked oxygen consumption, mitochondrial respiration, and ATP generation, increasing Glu, GSH, and nicotinamide adenine dinucleotide (NADH) levels, reducing the amounts of ROS to protect cells from H_2_O_2_-induced apoptosis [44]. GLS2 protected cells from DNA oxidation by preventing the generation of 8-hydroxy-2’-deoxyguanosine, the main source of oxidation-associated mutagenesis [45]. In various cell models, GLS2 knockout works as an anticancer factor, correlated with increased levels of ROS in several cancer types, i.e., neuroblastoma [46], cervical cancer [47], and triple-negative breast cancer (TNBC) [48]. Although GLS2 increased GSH levels in neuroblastoma, its expression correlated with the proliferation and aggressiveness of neuroblastoma by an N-MYC-dependent mechanism [46]. In cervical cancer, following radiotherapy, tissues of radiosensitive patients showed diminished GLS2 levels and their lower amounts of GLS2 were concomitant to decreased values of GSH, NADH, and nicotinamide adenine dinucleotide phosphate (NADPH), as well as higher oxidative stress. Hence, GLS2 was associated with the radioresistance of cervical cancer [47]. Of note, when GLS was either pharmacologically inhibited by CB-839 or genetically silenced, GLS2 mimicked its metabolic function, as well as its impact on redox balance, in a long number of TNBC cell lines [48]. Remarkably, after analyzing data from breast cancer tissues available from The Cancer Genome Atlas (TCGA), these authors found a correlation between higher GLS2 expression, increased epithelial-to-mesenchymal transition (EMT), and worse prognosis and mortality [48]. However, in a more in-depth study including 1075 patient samples from the TCGA database, higher GLS/GLS2 ratios were correlated with increased EMT in breast cancer patients and worse survival [49].

## 3. Mitochondrial Metabolism of Glutamine in Cancer: Redox Balance

Metabolic reprogramming is one of the hallmarks of cancer [50]. Metabolic adaptations are essential for providing the hugely increased bioenergetics and biosynthetic demands to sustain tumor growth [51] but also for maintaining an adequate oxidative–reductive equilibrium for cancer cells’ survival and proliferation [52]. Maximizing OXPHOS is among the metabolic traits most commonly altered in tumor cells [53]. In this metabolic shuffling, Gln is one of the most valuable playing cards [54]. Although Gln is not an essential amino acid, cancer cells usually promote its import and/or additional biosynthesis from other sources [55] through several oncogenes that activate glutaminolysis, i.e., c-MYC [56], KRAS [57], hypoxia-inducible factor 1 (HIF1) [58], isocitrate dehydrogenase 1 and 2 (IDH1/2) [59], epidermal growth factor receptor (EGFR) [60] or even tumor suppressor p53 [44]. Gln is the most abundant amino acid in circulating blood and in muscle [43]. Tumor cells can use Gln for biosynthetic purposes through the tricarboxylic acid cycle (TCA cycle) [61], allowing cancer cells to sustain TCA cycle activity and produce reductive equivalents such as NADPH during proliferation [62]. Additionally, it may be used to produce NADH, flavin adenine dinucleotide (FADH_2_), and ATP [63]. Oxidation of Gln equally sustains redox homeostasis by a reduction of NADP^+^ to NADPH by malic enzyme (ME1) due to the transformation of Gln-derived metabolites to pyruvate [54]. On the other hand, Gln is required for the synthesis of the main nonenzymatic antioxidant in the cell, the tripeptide GSH [64], which is synthesized from Glu, cysteine (Cys), and glycine (Gly) [5]. Glutamate production from glutamine is essential for GSH biosynthesis, since it not only provides one of the three amino acids needed but because produced glutamate may also serve to import cysteine to the cell through the xCT transport system; thus, GLS and GLS2 become key enzymes in cancer [3], contributing to the maintenance of redox homeostasis and the balance of ROS levels [65]. Additionally, the TCA cycle’s rewiring causes changes in the redox homeostasis, connecting the cancer metabolisms of amino acids and the redox biologies of tumor cells [66].

**Figure 1:**
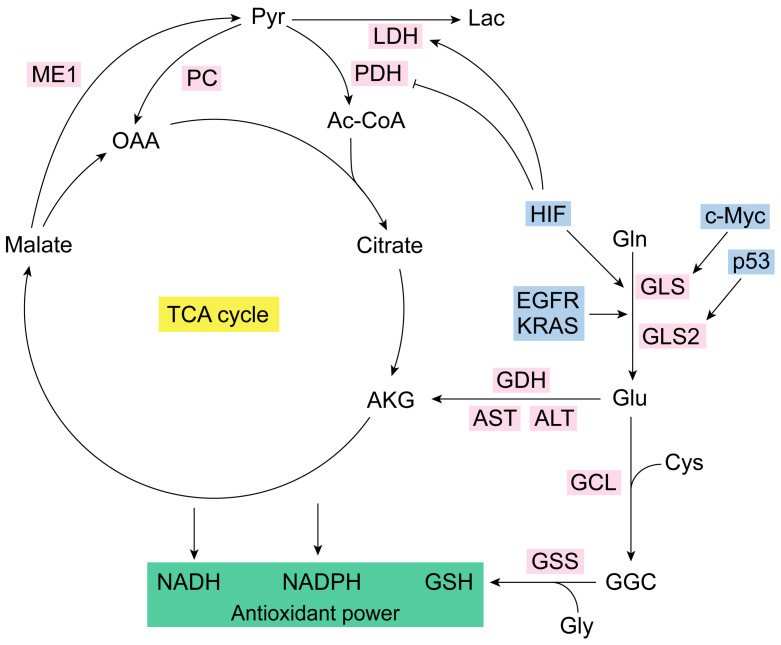
Glutaminolysis and antioxidant capacity. Glutaminase isoenzymes generate Glu from Gln [7]. GLS is regulated by the oncogene c-Myc, and GLS2 is modulated by tumor-suppressor p53 [44,45,56]. Glu together with Cys (via GCL) and Gly (via GSS) form, respectively, GGC and GSH (the most important antioxidant molecule in the cell) [1]. Glutaminolysis is accurately regulated [3,34]. IDH, KRAS, EGFR, and HIF are some of its key modulators [57,58,59,60]. HIF also controls LDH and PDH [70,71,72,73,74]. NADPH generation does not occur in the TCA cycle but in the IDH1/2 reaction that converts isocitrate to AKG [58]. Ac-CoA, acetyl coenzyme A; AKG, alpha-ketoglutarate; ALT, alanine aminotransferase; AST, aspartate aminotransferase; Cys, cysteine; EGFR, epidermal growth factor receptor; GCL, glutamate-cysteine ligase; GDH, glutamate dehydrogenase; GGC, gamma-glutamylcysteine synthetase; Gln, glutamine; GLS, glutaminase isoenzyme 1; GLS2, glutaminase isoenzyme 2; Glu, glutamate; Gly, glycine; GSH, glutathione; GSS, glutathione synthetase; HIF, hypoxia-inducible factor; Lac, lactate; LDH, lactate dehydrogenase; ME1, malic enzyme 1; NADH, nicotinamide adenine dinucleotide; NADPH, nicotinamide adenine dinucleotide phosphate; OAA, oxaloacetate; PC, pyruvate carboxylase; PDH, pyruvate dehydrogenase.

From a strict metabolic point of view, the catabolism of Gln enables cells to sustain enough anaplerotic flux to utilize a large number of TCA cycle intermediates as precursors for macromolecular biosynthesis [67]. Of note, Gln’s main role in many routes of intermediary metabolism that produce Glu and alpha-ketoglutarate (AKG) makes this amino acid an essential source of carbon for the TCA cycle [68]. Gln can be transformed to Glu by mitochondrial GA, which delivers Gln’s amide group as cationic-free ammonium (Figure 1). Glu can be turned to AKG by Glu dehydrogenase (GDH), which releases the amino group as a free ammonium ion, or by mitochondrial aminotransferases such as alanine aminotransferase (ALT) and aspartate aminotransferase (AST), which transfers the amino group of Glu to pyruvate or to oxaloacetate to form alanine or aspartate, respectively [5]. AKG enters the TCA cycle and through sequential reactions generates oxaloacetate (OAA) [67]. Then, Gln can be a substrate for the synthesis of both OAA or, subsequently, lactate, through glutaminolysis, which accumulates reductive capacity as NADH and NADPH [68]. Glutaminases are required by many cells to achieve maximal growth both in vitro and in vivo [69]. However, even Gln-addicted cells can reprogram their carbon metabolism from glucose to synthesize building blocks normally supplied by Gln [70]. The enzyme responsible for this form of glucose-dependent anaplerosis is pyruvate carboxylase (PC), fundamental for cell survival and proliferation of cells adapted to growth in low-Gln concentrations [58]. PC replenishes OAA and generates citrate through reductive carboxylation, facilitating an alternative anaplerotic pathway in Gln-independent tumor cells [71]. Silencing GLS in tumor cells increases PC activity to compensate for reduced anaplerosis from Gln, affecting cancer growth [72]. Gln-dependent anaplerosis is energetically advantageous as PC requires ATP to generate OAA, while GLS is not ATP-dependent [70]. Gln catabolism through the TCA cycle forms reducing equivalents (NADH) for OXPHOS [72]. Cancer cells can utilize the bidirectional metabolism from Gln-derived AKG to synthesize citrate and generate reducing equivalents, which is advantageous for adapting to nutrient availability [71].

### Glutaminase can Trigger Reductive Carboxylation in Cancer

The IDH1 isoform is located in the cytoplasm, whereas the IDH2 and IDH3 isoforms are found in mitochondria. IDH1 and -2 catalyze the decarboxylation of isocitrate into AKG, rendering NADPH, while IDH3 forms AKG, and NADH and CO_2_ form isocitrate [73]. The noncanonical process of the formation of isocitrate from AKG and CO_2_ is named reductive carboxylation, and it is fundamental in hypoxia conditions or when a high level of the synthesis of lipids is required [74]. Reductive carboxylation is catalyzed by IDH1/2 through the consumption of NADPH. Of note, NADPH and NADP^+^ cannot cross the mitochondrial membrane, a fact that implicates IDH1/2 in the balance of the oxidative status in cytosol and mitochondria [63,73,74,75]. IDH1 is related to the scavenging of ROS in the cytoplasm by the generation of NADPH in this cellular compartment [76]. In addition, the generation of cytosolic ROS mediates the induction of reductive carboxylation through IDH1 by negatively regulating a protein tyrosine phosphatase, which results in the increased phosphorylation of IDH1 and, hence, its activation [75].

**Figure 2:**
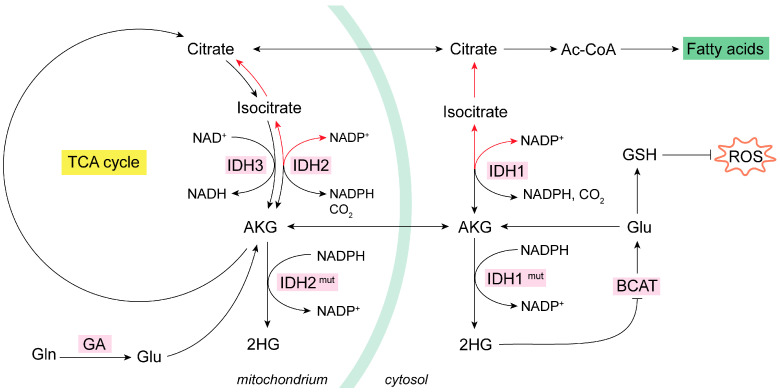
Mitochondrial oxidative and reductive metabolism of Gln. IDH isoenzymes modulate the oxidative or reductive metabolism of Gln in NADP-dependent reactions [74]. In cancer cells, the increase in glycolysis and glutaminolysis boost ROS levels [55]. In some cancer types, mutant IDHs produce oncometabolite 2HG, which inhibits BCAT isoenzymes and lowers GSH [77,78,79]. Many types of tumor cells activate the reductive metabolism (red arrows) of AKG to generate Ac-CoA for de novo lipogenesis as a response to their biosynthetic need for rapid proliferation [80,81,82,83,84]. Reductive carboxylation by IDH1 in the cytosol consumes NADPH to generate isocitrate and then citrate; subsequently, the citrate is imported into the mitochondria and there follows oxidative metabolism (black arrows) and decarboxylation via IDH2 to generate mitochondrial NADPH [73,74,75]. Ac-CoA, acetyl coenzyme A; AKG, alpha-ketoglutarate; BCAT, branched-chain amino acid aminotransferase; GA, glutaminase; GDH, glutamate dehydrogenase; GLS, glutaminase isoenzyme 1; GLS2, glutaminase isoenzyme 2; Gln, glutamine; Glu, glutamate; GSH, glutathione; HG, hydroxyglutarate; HIF, hypoxia-inducible factor; NADH, nicotinamide adenine dinucleotide; NADPH, nicotinamide adenine dinucleotide phosphate; OAA, oxaloacetate; ROS, reactive oxygen species.

There are tumor-associated IDH1/2 mutant versions that consume NADPH to transform AKG to the (*R*) enantiomer of 2-hydroxyglutarate, (*R*)-2HG, which accrues in cancer cells [73]. These (*R*)-2HG-producing mutants of IDH1/2 are commonly found in a subtype of gliomas [77]. (*R*)-2HG is considered an oncometabolite capable of inhibiting the branched-chain amino acid aminotransferases 1 and 2 (BCAT1/2), which use AKG and branched-chain amino acids as substrates to produce Glu and the corresponding branched-chain alpha-ketoacid, in a reversible amino group transfer reaction [78]. Hence, in this molecular context, the generation of Glu by transamination is compromised and becomes limiting for the biosynthesis of GSH [79], therefore impacting redox homeostasis (Figure 2). The activation of reductive carboxylation reprograms Gln metabolism, increasing GSH biosynthesis in a global compensatory rewiring [66]. This mechanism follows TCA cycle inhibition or impairment of the mitochondrial thioredoxin reductase (TrxR) antioxidant system [68]. Thus, to normalize antioxidant power, reductive carboxylation of Gln-derived AKG takes place [80]. The exchanging of glucose to Gln in the TCA cycle toward reductive Gln metabolism is conducted by HIFs in a mechanism regulated by the Warburg effect, also known as fermentative glycolysis [81]. For example, in normoxic 3D spheroids lung cancer cells, reductive citrate, produced by IDH1 in the cytosol from AKG derived from Gln is used to maintain redox homeostasis. The citrate generated in the cytosol was subsequently imported into the mitochondria, where an oxidative metabolism occurs via IDH2, forming NADPH, which can be used by various antioxidant systems to detoxify mitochondrial ROS [82]. Similarly, in H460 lung cancer cells, AKG’s reductive carboxylation allowed for the flow of citrate from the cytosol to the mitochondria, resulting in oxidative stress mitigation and faster growth rates [80]. Eventually, the increased consumption of Gln by reductive carboxylation of Gln-derived AKG supports anabolic reactions, such as lipid synthesis, to activate cell proliferation [83]. Reductive carboxylation of Gln-derived AKG was equally found in hepatoma cells and mouse xenograft models to support de novo lipogenesis and the cellular antioxidant system by increasing the levels of NADPH and GSH [84]. On the other hand, Gln-derived metabolites in the TCA cycle may serve for synthesizing aspartate, an essential metabolite for de novo nucleotide biosynthesis. In renal cell carcinoma, GLS inhibitor BPTES impaired the reductive transformation of Gln-derived carbons to aspartate and subsequently minimized the de novo synthesis of pyrimidines [85,86]. Accordingly, in three GBM cell lines treated with the more potent GLS inhibitor CB-839, a sharp drop in aspartate together with diminishments in both the oxidative metabolism in the TCA cycle and reductive carboxylation of Gln-derived AKG were found. These findings correlated with lower levels of metabolites from de novo purine and pyrimidine biosynthesis pathways [87]. In NSCLC, reductive carboxylation of AKG increased cytosolic ROS by a mechanism that includes the upregulation of NADPH oxidase 2 (NOX2) [75]. On the other hand, GLS inhibition by BPTES also evoked higher intracellular ROS levels by affecting GSH formation [85].

## 4. Glutaminases, Ferroptosis, and ROS

**Figure 3:**
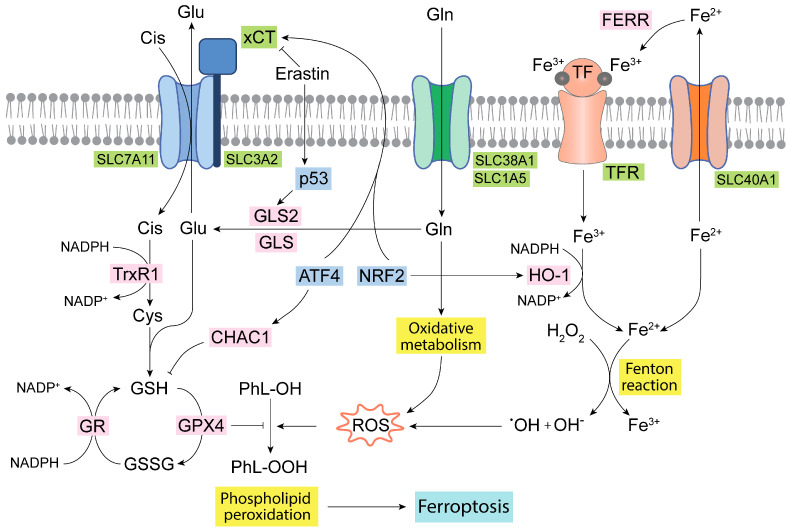
Ferroptosis and Gln homeostasis. Iron metabolism in the mitochondria generates oxidative molecules and radicals that together with the oxidative metabolism of Gln produce a large amount of ROS, leading to lipid peroxidation and ferroptosis [41,98]. The glutathione antioxidant system formed by GR, GPX, and TrxR isoenzymes, as well as by GSH, is the main antioxidant network to detoxify ROS [90,97]. Some transcription factors, such as ATF4 and NRF2, regulate and connect the GSH antioxidant system, Glu transport, and iron metabolism [29,96]. ATF4, activating transcription factor 4; CHAC1, ChaC glutathione specific gamma-glutamylcyclotransferase 1; Cis, cystine; Cys, cysteine; FERR, ferritin; GLS, glutaminase isoenzyme 1; GLS2, glutaminase isoenzyme 2; Gln, glutamine; Glu, glutamate; GPX4, glutathione peroxidase 4; GR, glutathione reductase; GSH, reduced glutathione; GSSG, oxidized glutathione; HO-1, heme oxygenase 1; NADPH, nicotinamide adenine dinucleotide phosphate; NRF2, nuclear erythroid factor 2-related factor 2; PhL-OOH, peroxidized phospholipids; PhL-OH, phospholipids; ROS, reactive oxygen species; TF, transferrin; TFR, transferrin receptor; TrxR1, thioredoxin 1; xCT, cystine/glutamate antiporter.

Ferroptosis is an iron-dependent specific and regulated type of cell death. The mechanism is mainly characterized by high levels of intracellular iron, which through the Fenton reaction leads to the production of hydroxyl radicals, subsequently provoking polyunsaturated fatty acid peroxidation, lastly resulting in membrane disruption and cell death [88]. Glutamate, which is produced by GLS and GLS2, is essential for the synthesis of GSH, which reduces lipid hydroperoxides via glutathione peroxidase 4 (GPX4) [89]. Thus, a decrease in GSH levels can lead to an increase in the lipid peroxidation process, and, therefore, the ferroptosis phenomena, because GSH is the most important nonenzymatic antioxidant and serves as a fundamental eliminator of ROS [88]. GPX4 may act as an oncogene, as it works as the major inhibitor of ferroptosis, also being related to the regulation of the electron transfer chain (ETC), OXPHOS, and other metabolic pathways in cancer [89]. Noteworthy, erastin, an inhibitor of the xCT cystine-Glu antiporter, is another fundamental regulator of ferroptosis (Figure 3). An induction of ferroptosis by erastin evokes GSH depletion by limiting the cysteine supply [90]. Erastin-dependent induction of ferroptosis is impacted by the mitochondrial metabolism of Gln, as it has been found that the reductive carboxylation of Gln-derived metabolites favors resistance in NSCLC cells via a redox mechanism involving the GSH antioxidant system [91]. GA isoenzymes have a profound link with ferroptosis, since they are fundamental enzymes for achieving a high glutaminolytic flux in cancer cells [81]. Hence, ferroptosis is modulated by the TrxR system upon erastin action, as well as by glutaminolysis [90]. Thus, a connection between Gln metabolism and ferroptosis via the GLS2-p53 axis has been demonstrated [92,93]. Mechanistically, p53 inhibited the transcription of the cystine/Glu antiporter SLC7A11 (system xCT) [94]. Additionally, in ccRCC, GLS2 was upregulated during ferroptosis induced by erastin or RSL3. GLS2 is related to higher amounts of GSH in the cell and is considered a potential ferroptosis suppressor in ccRCC [95]. Ferroptosis was equally activated in human TNBC cells in a mechanism dependent on the action of the activating transcription factor 4 (ATF4) and ChaC glutathione-specific gamma-glutamylcyclotransferase 1 (CHAC1), which degrades GSH and induces cystine deprivation [96]. Mechanistically, this might result in the relocation of reducing equivalents from cystine to thioredoxin (via TrxR1) and thereby avoids ROS augmentation [97]. Although GSH prevents the interaction of iron, oxygen, and polyunsaturated fatty acids from triggering ferroptosis, the exact role of total GSH (tGSH) in ferroptosis and whether there exist differences between cytosolic and mitochondrial GSH for the induction of ferroptosis are still to be clarified [98].

Ferroptosis modulation is essential in the chemoresistance event in TNBC [99], whereby tumor cells are characterized as commonly being Gln-dependent via glutaminolysis, which favors drug resistance [100]. To defeat these cancer cells, a dual combination strategy has been suggested by targeting both GLS and ferroptosis [99]. Gallbladder cancer cells, which also need GLS for growth and proliferation, triggered ferroptosis following GLS inhibition and a drop in antioxidant capacity (GSH- and NADPH-dependent), which evoked oxidative stress [101]. Ovarian cancer cells are enriched in iron and rewire their Gln metabolism through HIF-mediated metabolic reprogramming [102]. However, ovarian cancer cells are resistant to ferroptosis through NRF2-mediated activation of additional antioxidant mechanisms, as those elicited by ferritin and heme oxygenase-1 (HO-1) decrease free iron amounts, modulating iron metabolism and ROS levels [103]. In these conditions, therapeutic strategies that either block the xCT system or target specific metabolic vulnerabilities may be an option to overcome ferroptosis resistance [102]. In several types of aggressive and metastatic cancers a combination treatment including the natural product beta-elemene, in a codelivery strategy, was an efficacious drug for tumor patients reversing multidrug resistance and activating ferroptosis [104]. In KRAS-mutant CRC cells, a combination of beta-elemene and cetuximab was successful, both in vitro and in vivo, and resulted in the induction of ferroptosis via the downregulation of GLS, xCT, GPX4, ferritin, and ferroportin (SLC40A1), which exports intracellular iron out of the cell [105].

## 5. Future Perspectives

As reported above, the GLS and GLS2 isoenzymes are metabolic enzymes [42,43,54,63] whose roles may imply key antioxidant functions [64,65,91,95], which might lead to therapeutic implications for cancer therapy [15,16,26,35]. Of interest, several GLS inhibitors can block cancer progression by promoting oxidative stress [106]. Thus, a novel derivative of lonidamine, named HYL001, greatly inhibited cancer stem cells (CSCs) by hampering Gln metabolism, with 380-fold and 340-fold lower IC_50_ against breast CSCs and hepatocellular carcinoma (HCC) stem cells, respectively, as compared to lonidamine, while having minimum toxic effects toward nontumor cells and immune-competent mice [13]. Although lonidamine increased ROS and promoted cell death by reduction in the pentose phosphate pathway (which lowered NADPH and GSH formation) [107], it is not approved in the clinic due to its hepatotoxicity, which is caused by its low solubility and pharmacokinetic characteristics [108]. However, HYL001 constitutes a promising derivative and is confirmed to be a good candidate for clinical trials, such as through reduced cancer cell proliferation on fresh tumor tissues from HCC patients by diminishing GSH, increasing ROS levels, and inducing apoptosis [13]. Similarly, Gln metabolism and, particularly, GLS activity were found to be essential for androgen-receptor antagonist-resistant prostate cancer cells, xenografts, patient-derived organoids, patient-derived explants, and tumor samples by increasing antioxidant capacity [106].

Cancer is extremely adaptive with compensatory metabolic pathways that inevitably ease resistance to Gln shortages, specifically with monotherapy [109]. Blocking GLS by CB-839 or genetic silencing made resistant cancer cells vulnerable in an antioxidant-dependent mechanism, which included GSH action [106]. However, because of the plasticity of tumors and heterogeneity in cancer, it is not simple to target their vulnerabilities, as normal and malignant cells share many essential enzymes, metabolic network pathways, and antioxidant systems [52]. Additional efforts are vital to delimitate how to modulate the redox status against every type of cancer and optimize combinations for synergistic therapy, hopefully to achieve personalized successful treatments. For example, because poly (ADP-ribose) polymerase (PARP) inhibitors synergize with GLS ones [85], a phase II clinical trial was started to explore the consequences of the association of CB-839 with a PARP inhibitor in metastatic cancer prostate cells (NCT04824937). Ferredoxin 1 (FDX1) is another targetable antioxidant system because of its key function in the mitochondrial membrane of tumor cells with an impaired ETC [110]. Resistant pancreas cancer cells were also sensitive to an FDX1-selective drug, which increased ROS levels and decreased cell viability [106]. A compensatory dual strategy targeting the GLS-associated antioxidant program might be reinforced by a ferredoxin inhibition and/or a ferroptosis activation to provide novel therapeutic tools [91]. Thus, targeting the xCT system, ferroptosis and GLS in a combination therapy, consisting of erastin + doxorubicin + CB-839, has shown promising results in TNBC, tumor cells very sensitive to CB-839 treatment, because its high reliance on Gln and elevated GLS expression [100]. Other efforts to synergistically cooperate with both GLS silencing and ROS enhancement might span MYC, and the PTEN/PI3K/mTOR axis [109], as well as xCT, GPX, and glucose transporters (GLUTs) [102]. Interestingly, disrupting both cellular redox balance and Gln availability by erastin and CB-839 has produced a positive synergistic effect in cisplatin therapy, sensitizing chemo-resistant TNBC cells [100]. These findings offer promising clinical relevance and deserve additional in-depth studies.

## Figures and Tables

**Table 1:** GLS in the control of redox status in cancer.

Molecular Mechanism(s)/Impact on Cancer	Cancer Type(s)	In Vitro Model(s)	In Vivo Model(s)	Reference
NRF2 ^1^ caused tumor growth through a mechanism that includes GLS overexpression	Breast	BT474, SKBR3	Nude mice in orthotopic models	[8]
Gln-independent cells lowered GLS, increased oxidative stress, and enhanced resistance to drugs undergoing EMT ^2^	Breast (TNBC ^3^)	MDA-MB-231, SUM149, 4T1	Female athymic nude xenografted mice	[9]
GLS inhibition by CB-839 synergistically worked with the inhibition of the ERRα ^4^, blocking NADPH ^5^ synthesis and decreasing tumor growth	Breast (TNBC ^3^)	MDA436	None	[10]
Iron oxidative nanoparticles coupled to the GLS inhibitor CB-839 both increased ROS and decreased GSH, boosting DNA oxidative damage and cancer cell death	Breast (TNBC ^3^)	MDA-MB-231	Mice injected with tumor cells and iron-CB-839 nanoparticles	[11]
AMPK ^6^ activated NRF2 ^1^ and its target proteins to allow tumor cells to grow and maintain their redox status through GLS, supporting anchorage-independent cancer cell survival	TNBC ^3^, Liver, Pancreas, Skin	MDA-MB-231, HepG2, BxPC-3, HT-1080, HaCaT	None	[12]
HYL001, a new drug with a low IC_50_ against cancer cells and minimal toxicity toward normal cells and healthy mice, repressed GLS, reduced GSH, enhanced ROS, and blunted the TCA ^7^ cycle and OXPHOS ^8^	TNBC ^3^, Liver (HCC ^9^)	4T1, H22	4T1 metastatic and orthotopic models in BALB/c mice	[13]
Induction of ARHI ^10^ resulted in oxidative stress, which was augmented following GLS inhibition by BPTES	Ovarium	SKOv3	Xenografted mice bearing SKOv3 cells	[14]
BPTES prevents the interaction between NQO2 ^11^ and caveolin-1 of cancer cells that induce their metastatic activity	Prostate	LNCaP, C4, C4-2	None	[15]
Inhibition of GLS increased DNA oxidative damage and boosted susceptibility to ionizing radiation	Prostate	DU145, LNCaP	PC3 injected into nude NSG ^12^ mice	[16]
Mutant G6PD ^13^ melanoma cells increased glutaminolysis, which correlated with higher ROS levels, decreased NADPH, and lower GSH/GSSG ratios	Skin (melanoma)	M481, M214, A375	Melanoma cell lines injected into nude NSG ^11^ mice	[17]
GLS inhibition by CB-839 increased mitochondrial ROS, lowered the GSH/GSSG ratio, enhanced apoptosis, and diminished cancer growth in vitro and in vivo	Colon (CRC ^14^)	HCT116, C26	CRC ^14^ in BALB/c mice and CRC ^14^ from patients in NSG ^12^ mice	[18]
KRAS-mutant cells increased sensitivity to GLS inhibition by CB-839 through NRF2 ^1^	Pancreas	BxPC3, Panc-1, MiaPaC2	None	[19]
Oxidative stress increased glutaminolysis and the production of NADPH ^5^ and GSH	Pancreas (PDAC ^15^)	SW1990	Nude BALB/c mice	[20]
GLS succinylation was essential to maintain redox homeostasis measured as NADPH and GSH levels, as well as ROS formation	Pancreas (PDAC ^15^)	SW1990	Male athymic nude BALB/c mice	[21]
Lactate imported by MCT1 ^16^ maintained redox homeostasis via NRF2 ^1^ and thereby cell viability following GLS inhibition by CB-839, which shortened GSH and increased ROS	Pancreas (PDAC ^15^)	T3M4, A818-6	PDAC ^15^ patients with a tumor disease at stage T3N1M0	[22]
Following GLS inhibition by BPTES or CB-839, cancer cells showed decreased survival and more apoptosis associated with a lowered GSH/GSSG ratio, increased NRF2 ^1^, and higher oxidative DNA damage	Kidney	SN12, 786-O	Mice with orthotopic injections and treated with CB-839	[23]
HSP60 silencing activated the MEK/ERK/c-MYC axis to evoke Gln addiction while increasing susceptibility to oxidative stress and GLS inhibition by BPTES	Kidney	786-O, 769-P	None	[24]
More aggressive tumors showed higher GLS activity, increased ROS levels, enhanced GSSG/GSH ratios, and accumulation of NAD^+^ and NADP^+^	Thyroid	B-CPAP, K1, TPC-1	None	[25]
GLS inhibition by CB-839 induced oxidative stress, i.e., lowering GSH/GSSG, enhancing TrxR1 ^17^, and diminishing tumor growth	Uterine, Cervix	CaSki, SiHa	Nude mice were xenografted with SiHa cells	[26]
CB-839 in combination with radiation increased oxidative stress and boosted DNA oxidative damage	HNSCC ^18^	CAL-27, FaDu, HN5	Nude mice xenografted with CAL-27/HN5	[27]
*Keap1*-mutant cells displayed a robust sensitivity to GLS inhibition by BPTES and CB-839 through NRF2 ^1^, increasing survival ratios	Lung	Human adenocarcinomas	Mice xenografted, treated with CB-839	[28]
Oxidative stress, by NRF2 ^1^ malfunction, depleted Glu, which was lowered by GLS inhibition by CB-839, blocking cancer growth	Lung	LKR10/13	Mice subcutaneously injected with tumor cells	[29]
Selenite impaired GLS expression and increased the GSSG/GSH ratio	NSCLC ^19^	A549	Ex vivo NSCLC ^18^	[30]
TGFβ ^20^ induced EMT ^2^ that evoked sensitivity to BPTES, which reduced citrate levels and OXPHOS ^8^, lowering the cells’ antioxidant capacity	NSCLC ^19^	A427, NCI-H358	None	[31]
Lower levels of NADH, GSH, and GSSG were concomitants to longer survival after using a combination of an HDAC6 ^21^ inhibitor + CB-839 in in vitro and in vivo KRAS/LKB1 ^22^ models (displaying high GLS activity)	NSCLC ^19^	H23, H358	C57BL/6 mice inoculated with KRAS/TP53 or KRAS/LKB1 cancer cells	[32]
Activation of SAT1 ^23^ increased GLS activity and GSH synthesis, ameliorating oxidative stress to support lung cancer cell proliferation, which was blocked by inhibition of GLS, resulting in ROS accumulation	NSCLC ^19^	A549, PC9, H1650, H1792, H358, H1944	PC9 cells were subcutaneously injected into BALB/c nude mice	[33]
GAC isoform was highly expressed versus KGA isoform in GBM ^24^ and more malignant astrocytomas	Brain	U87MG	Astrocytomas of different malignancies	[34]

^1^ NRF2, nuclear factor erythroid 2-like 2; ^2^ EMT, epithelial-to-mesenchymal transition; ^3^ TNBC, triple-negative breast cancer; ^4^ ERRα, estrogen-related receptor alpha; ^5^ NADPH, nicotinamide adenine dinucleotide phosphate; ^6^ AMPK, adenosine monophosphate-activated protein kinase; ^7^ TCA, tricarboxylic acid; ^8^ OXPHOS, oxidative phosphorylation; ^9^ HCC, hepatocellular carcinoma; ^10^ ARHI, aplasia Ras homolog member I (also named DIRAS3); ^11^ NQO2, nicotinamide riboside:quinone oxidoreductase; ^12^ NSG, NOD-scid IL2Rgammanull; ^13^ G6PD, glucose 6-phosphate dehydrogenase; ^14^ CRC, colorectal cancer; ^15^ PDAC, pancreatic ductal adenocarcinoma; ^16^ MCT1, monocarboxylate transporter-1; ^17^ TrxR1, thioredoxin reductase 1; ^18^ HNSCC, head and neck cancer squamous cell carcinoma; ^19^ NSCLC, non-small-cell lung carcinoma; ^20^ TGFβ, transforming growth factor beta; ^21^ HDAC6, histone deacetylase 6; ^22^ LKB1, liver kinase B1; ^23^ SAT1, spermidine/spermine N1-acetyltransferase 1; ^24^ GBM, glioblastoma.

**Table 2:** GLS2 in the control of redox status in cancer.

Molecular Mechanism(s)/Impact on Cancer	Cancer Type(s)	In Vitro Model(s)	In Vivo Model(s)	Reference
Inhibition of GA by the compound 968 in apigenin-treated cells decreased NADPH and increased intracellular ROS levels, boosting apoptosis	Lung	H1299, H660	None	[35]
*GLS* silencing and *GLS2* overexpression induced oxidative stress, increased apoptosis, and decreased cell migration	Brain (GBM ^1^)	SFxL, LN229, T98G	None	[36]
Silencing of *GLS* or overexpression of *GLS2* decreased oxidative status and boosted antioxidant enzymes	Brain (GBM ^1^)	LN229, T98G	None	[37]
GLS2 reduced the TMZ ^2^ resistance of GBM ^1^ in vitro and in vivo through the long noncoding RNA ATXN8OS, which mediated ferroptosis and increased oxidative damage to lipids	Brain (GBM ^1^)	U251, U251TR	U251 + GLS2-transfected cells were injected into the brains of nude mice	[38]
p73 Transcriptionally activated GLS2, increased serine and diminished oxidative stress	NSCLC ^3^, Osteosarcoma	H1299, SaOs-2	None	[39]
GLS2 overexpression shortened oxidative stress by a GSH-independent mechanism	NSCLC ^3^	CL1-0	None	[40]
Knockout of GLS2, a tumor suppressor in this study, reduced the GSH/GSSG ratio and increased oxidative damage to lipids during ferroptosis	Liver (HCC ^4^)	HepG2, HepG3, SKHep1	Injections of SKHep1 cells on the flanks of NSG ^5^ mice	[41]

^1^ GBM, glioblastoma; ^2^ TMZ, temozolomide; ^3^ NSCLC, non-small-cell lung carcinoma; ^4^ HCC, hepatocellular carcinoma; ^5^ NSG, NOD-scid IL2Rgammanull.

## Data Availability

Data are contained within the article.

## Abbreviations

AKG, alpha-ketoglutarate; ALT, alanine aminotransferase; AST, aspartate aminotransferase; ATF4, activating transcription factor 4; ATP, adenosine triphosphate; BCAT1/2, branched chain amino acid aminotransferases 1 and 2; ccRCC, clear cell renal cell carcinoma; CHAC1, ChaC glutathione specific gamma-glutamylcyclotransferase 1; CSC, cancer stem cell; Cys, cysteine; EGFR, epidermal growth factor receptor; EMT, epithelial–mesenchymal transition; ESCC, esophageal squamous cell carcinoma; ETC, electron transfer chain; FADH2, flavin adenine dinucleotide; FDX1, ferredoxin 1; GA, glutaminase; GAB, longer GLS2 transcript and isoform; GAC, shorter GLS transcript and isoform; GBM, glioblastoma; GDH, glutamate dehydrogenase; Gln, glutamine; GLS, glutaminase isoenzyme; GLS2, glutaminase isoenzyme 2; Glu, glutamate; GLUT, glucose transporter; Gly, glycine; GR, glutathione reductase; GSH, reduced glutathione; GSSG; oxidized glutathione; GPX4, glutathione peroxidase 4; HCC, hepatocellular carcinoma; HIF1, hypoxia-inducible factor 1; HNSCC, head and neck cancer squamous cell carcinoma; HO-1, heme oxygenase-1; IDH1, isocitrate dehydrogenase 1; KGA, longer GLS transcript and isoform; LGA, shorter GLS2 transcript and isoform; mTOR, mammalian target of rapamycin; NADH, nicotinamide adenine dinucleotide; NADPH, nicotinamide adenine dinucleotide phosphate; NOX2, NADPH oxidase 2; NRF2, nuclear erythroid factor 2-related factor 2; NSCLC, non-small-cell lung carcinoma; OAA, oxaloacetate; OXPHOS, oxidative phosphorylation; PARP, poly(ADP-ribose) polymerase; PC, pyruvate carboxylase; PDAC, pancreatic ductal adenocarcinoma; PI3K, phosphatidylinositol-3-kinase; PTEN, phosphatase and tensin homolog; ROS, reactive oxygen species; TCA, tricarboxylic acid; TCGA, The Cancer Genome Atlas; tGSH, total glutathione; TNBC, triple-negative breast cancer.
